# The extinct, giant giraffid *Sivatherium giganteum*: skeletal reconstruction and body mass estimation

**DOI:** 10.1098/rsbl.2015.0940

**Published:** 2016-01

**Authors:** Christopher Basu, Peter L. Falkingham, John R. Hutchinson

**Affiliations:** 1 Structure and Motion Laboratory, Royal Veterinary College, Hawkshead Lane, North Mymms, Hatfield, Hertfordshire AL9 7TA, UK; 2 School of Natural Sciences and Psychology, Liverpool John Moores University, James Parsons Building, Bryon Street, Liverpool L3 3AF, UK

**Keywords:** *Sivatherium*, giraffid, body mass, photogrammetry, scaling, volumetric model

## Abstract

*Sivatherium giganteum* is an extinct giraffid from the Plio–Pleistocene boundary of the Himalayan foothills. To date, there has been no rigorous skeletal reconstruction of this unusual mammal. Historical and contemporary accounts anecdotally state that *Sivatherium* rivalled the African elephant in terms of its body mass, but this statement has never been tested. Here, we present a three-dimensional composite skeletal reconstruction and calculate a representative body mass estimate for this species using a volumetric method. We find that the estimated adult body mass of 1246 kg (857—1812 kg range) does not approach that of an African elephant, but confirms that *Sivatherium* was certainly a large giraffid, and may have been the largest ruminant mammal that has ever existed. We contrast this volumetric estimate with a bivariate scaling estimate derived from *Sivatherium's* humeral circumference and find that there is a discrepancy between the two. The difference implies that the humeral circumference of *Sivatherium* is greater than expected for an animal of this size, and we speculate this may be linked to a cranial shift in centre of mass.

## 1. Introduction

### (a) Taxonomy and morphology of *Sivatherium giganteum*

The Giraffidae clade is represented today by two extant species: *Giraffa camelopardalis*, well known for its large size and highly derived body proportions; and the smaller, more modestly proportioned *Okapia johnstoni.* The fossil record contains phenotypes that demonstrate progressive neck and limb elongation as one approaches the condition of extant *Giraffa* [1].

An alternative evolutionary pattern is displayed by the Sivatheriinae [2], a giraffid outgroup that first appeared in East Africa in the Late Miocene [3]. The type species, *Sivatherium giganteum*, is the Asiatic form, found near the Plio–Pleistocene boundary of the Himalayan foothills [4]. *Sivatherium giganteum* possessed apomorphic skeletal anatomy which was unique in the giraffid lineage, some of which is no longer represented in extant giraffids; the key features included a relatively short neck, short and thickened distal limbs, and ornate cranial appendages. For simplicity, we will refer to giraffid species by their genus names throughout this paper.

*Sivatherium* was initially misidentified as an archaic link between modern ruminants and the now obsolete, polyphyletic ‘pachyderms’ (elephants, rhinoceroses, horses and tapirs). The confusion arose in part due to the graviportal (robust) morphology, which was unlike anything else studied at that time. On the basis of the holotype specimen, a well-preserved skull, the body mass of *Sivatherium* has been anecdotally compared with that of the African elephant *Loxodonta africana* [5,6], which can weigh over 6500 kg [7]. To date, there has been no robust analysis to test this comparison.

Despite being such a peculiar mammal, *Sivatherium* remains infrequently studied beyond descriptive papers [5,8–10], where descriptions of the overall form vary. Functional analyses are even scarcer, with little added to the literature since the thoughtful commentary of Falconer and Cautley in the mid-1800s [5]. Informed analysis of this enigmatic animal will add to our overall understanding of the diversity that existed in the giraffid lineage.

There has been no rigorous reconstruction of the entire skeleton of *Sivatherium*. Here, we present a three-dimensional composite skeletal reconstruction based upon the originally described material held at the Natural History Museum, London (NHMUK). We then use this model to calculate a representative body mass estimate for this species, employing and comparing volumetric estimates with bivariate scaling estimates from skeletal measurements. In doing so, we provide an updated, modern scientific view of the shape and size of this long-neglected giraffid.

### (b) Body mass estimates in extinct taxa

Estimates of body mass are an indicator of important ecological traits such as metabolic rate, behaviour and reproduction [11]. The conventional method of estimating body mass in extinct animals is to use a bivariate (or, less commonly, multivariate) scaling relationship derived from a group of ecologically/morphologically similar animals [12]. This is usually in the form of a linear or log-transformed regression equation, typically using one or two skeletal measurements as the independent variable(s).

A previous discussion suggested that *Sivatherium* exceeds a theoretical upper size limit for ruminant mammals and used bivariate scaling equations to predict the body mass of African *Sivatherium* spp. from total skull length, metapodial width and dental measurements [13]. The wide range of resulting estimates (1230–3720 kg, a threefold difference) highlights the ‘one bone’ problem, where the resulting body mass estimate is highly sensitive to the choice of bone used.

Recent efforts in predicting body mass of other extinct taxa have illustrated the limitations of bivariate scaling methods and have instead adopted a volumetric approach [14,15]. With volumetric approaches, body mass estimates are calculated using the overall form of the animal versus an isolated metric. Recent studies have used the minimum convex hull method to calculate a body volume, where a surface is mathematically ‘shrink-wrapped’ around the extremities of the skeleton's functional segments. A body mass estimate can be calculated from the volume, either by assigning an assumed overall tissue density [16] or by using a predictive scaling equation [17].

## 2. Material and methods

### (a) Skeletal reconstruction

We created three-dimensional models of 26 fossilized bones from NHMUK assigned to *Sivatherium*, using Agisoft Photoscan photogrammetry software. All the bones used were skeletally mature; further details are documented in the electronic supplementary material. The resulting three-dimensional bone models were articulated using Autodesk Maya software. Most of the skeletal elements articulate together well; indeed, much of the postcranial material may represent one individual [18]. Apparent size variation between adjacent bones suggests the presence of at least three individuals in this collection. Two *Sivatherium* fossils required geometric scaling in order to articulate with adjacent elements: the metatarsus (scaled up by 4%) and the distal femur fragment (scaled down by 15%).

There are a number of skeletal elements for *Sivatherium* that have not yet been recovered, to our knowledge. Most of the non-cervical vertebral series is missing, as are a complete mandible, ribcage, pelvis and femoral diaphysis. The distal phalanges are also currently missing, although a distal phalanx was reportedly present in this collection [19].

Femur length was estimated from the available humeral length, using a quadrupedal mammal scaling model [7]. The distal phalanges have been scaled up by a factor of 1.5, from the distal phalanx of an extant giraffe hindlimb (detailed in the electronic supplementary material). This is the most reasonable choice, as the morphology of the homologous *Sivatherium* distal phalanx ‘exactly agrees’ with that of *Giraffa* [19].

**Figure 1.**
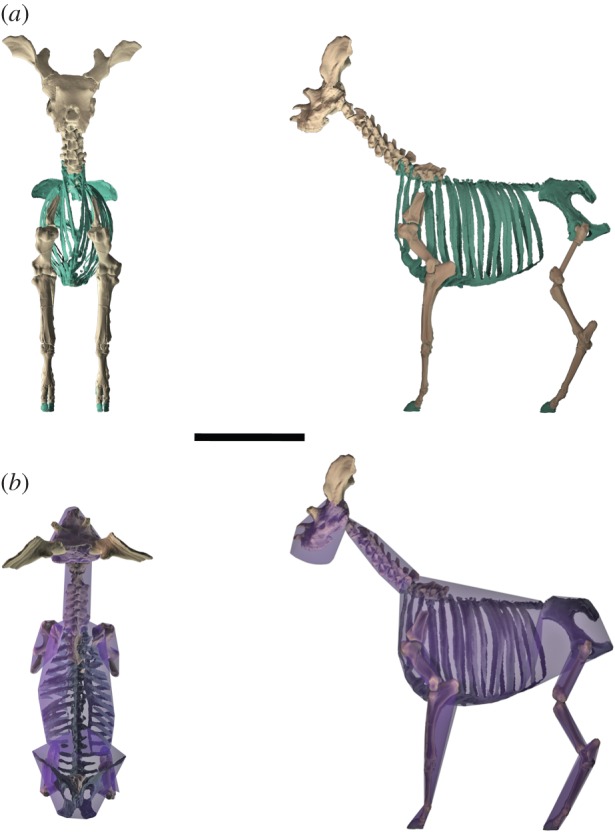
(*a*) Skeletal reconstruction of *S. giganteum*, cranial and lateral orthogonal views. Anatomy modelled from *Giraffa* is shown in green. (*b*) Skeleton with minimum convex hull in dorsal and lateral view. Black scale bar is equal to 1 m. (Online version in colour.)

A solution to the missing ribs, non-cervical vertebrae and pelvis is to model the entire torso from *Giraffa*, which assumes that thorax dimensions are conserved across Giraffidae, an assumption that is supported by three observations detailed in the electronic supplementary material. We have therefore modelled the *Sivatherium* torso from a giraffe skeleton point cloud [16], and geometrically scaled the giraffe torso to match the dimensions of available *Sivatherium* thoracic vertebrae (figure 1*a*). This again required a linear scaling factor of 1.5.

### (b) Body mass estimation

We estimated the body mass of an adult *Sivatherium* using humeral circumference [7] and convex hull volume [16,17]. As in previous studies of large extinct animals [14,15], we expected there to be a discrepancy between volumetric and skeletal estimates. We have also used thoracic circumference to make an estimate of body mass [20], as partial validation of the scaled-up *Giraffa* torso.

The skeleton was partitioned into functional segments (figure 1*b*), and we used the convex hull function in MeshLab to assign volumes to these segments. An additional volume was assigned to approximate the missing mandible. The total volume is calculated as the sum of the individual segment volumes.

**Figure 2.**
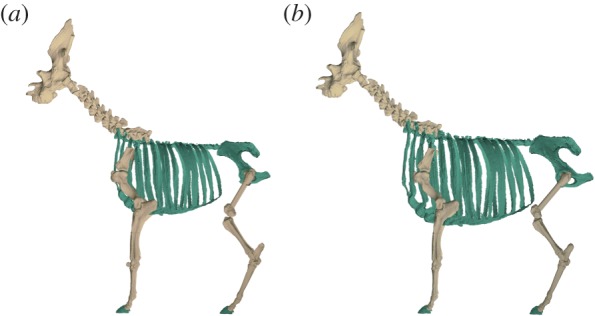
(*a*) ‘Minimum’ sensitivity analysis (of torso/femur size) model and (*b*) ‘maximum’ sensitivity analysis model. (Online version in colour.)

We performed a sensitivity analysis to evaluate the effect of uncertainty with respect to the initial modelled torso and femur, where the linear dimensions of these were increased and decreased by 10%. This resulted in ‘minimum’ and ‘maximum’ models (figure 2), which we consider to be realistic extremes of body proportions. We calculated volumetric estimates of body mass for these additional models and compared them with the initial reconstruction.

## 3. Results

The two volumetric calculations yielded similar results. As expected, there was a discrepancy between the volumetric predictions and the humeral circumference scaling method, although the prediction interval ranges did overlap (table 1). The minimum and maximum models yielded mass estimations of 1041 kg (719–1506) and 1533 kg (1048–2243), respectively.

**Table 1.** Body mass predictions for *S. giganteum*, with associated body mass prediction intervals, percentage prediction error (PPE) and *R*^2^ for each method used. Convex hull estimate using Sellers *et al*. [16] assumes a mean body density of 893.36 kg m^−3^. Further details are in the electronic supplementary material.

method	model	estimated mass (kg)	95% prediction interval (kg)		PPE	*R*^2^
			lower	upper		
convex hull	Brassey *et al*. [17]	1246	857	1812	11.6	0.976
	Sellers *et al*. [16]	1101	716	1487	34	0.975
humeral circumference	Campione & Evans [7]	3053	1578	5910	23.7	0.986
thoracic circumference	De Esteban-Trivigno & Köhler [20]	1966	1369	2824	13.5	0.980

## 4. Discussion

Both volumetric calculations show agreement in the resulting estimates (table 1), highlighting that either is an appropriate use of the convex hull volume. They also overlap with the body mass prediction interval derived from the thoracic circumference [20]. We now focus on the volumetric scaling method [17] and humeral circumference method [7]. For purpose of discussion, we assume that the estimate of 1246 kg represents the most plausible estimate of body mass, given the relatively low percentage predictive error (PPE) of the volumetric method (table 1), and the advantages of using a full body reconstruction versus a single bone.

The humeral circumference predicts that *Sivatherium* weighed approximately 3000 kg, over twice the estimate from the convex hull scaling method. The predictive intervals overlap towards the lower range of the humeral estimate owing to the relatively large PPE of this model compared with the volumetric model (table 1). Despite this overlap, the estimate derived from humeral circumference occupies a range of body masses that are conspicuously heavier than the volumetric estimate. The humeral circumference might overestimate body mass owing to random deviation from the scaling model, but alternatively this raises functional questions about the humeral morphology of *Sivatherium.* The discrepancy implies that on average the humerus has a larger circumference than expected for an animal of this size. The morphology of other forelimb bones in *Sivatherium* is consistent with this finding. For example, compared with most other giraffids (including extinct forms), the metacarpus is markedly thicker and relatively shorter [2].

An explanation for a robust forelimb could be a cranial shift in the centre of mass compared with other giraffids. Such a shift may be due to the presence of heavy cranial appendages. This in turn may be correlated with allometric thickening of the forelimb skeleton though phylogeny or ontogeny, similar to the suggestions made for ceratopsian dinosaurs, which possessed enlarged cranial crests [21].

Our estimate of *Sivatherium*'s body mass does not take into account the presence of the large cranial appendages, which were exclusively possessed by males [2,9]. Sexual dimorphism is seen in many large ungulates, including *Giraffa*, where adult males are larger than females (with mean masses of 800 kg for females and 1200 kg for males) [22]. The large distal femur described in ‘Material and methods’ was scaled down by 15% to fit with the rest of the hindlimb skeleton, indicating the presence of a larger individual in the same locality. We therefore deem that our current body mass estimate reflects the lower end of the potential size range. This suggests that *Sivatherium* surpassed extant *Giraffa* in terms of body mass and may have been the largest ruminant mammal that has ever existed.

Our sensitivity analysis also shows that the body mass estimate is sensitive to uncertainty in the thoracic and femoral dimensions. The resulting prediction intervals of the small, preferred and large models show a high degree of overlap; we therefore do not consider this sensitivity to be functionally significant.

## 5. Conclusion

This is the first time that modern quantitative methods have been applied to this understudied and morphologically bizarre mammal. *Sivatherium giganteum* did not weigh as much as an adult African (or even Asian) elephant, but certainly was a large giraffid, and may have been the largest ruminant mammal that has ever existed. The current body mass estimate of 1246 kg (857–1812 kg) likely reflects the lower end of the expected species variation, because this does not take into account the large posterior cranial appendages of male individuals, nor the presence of larger skeletal material within the same collection. The description and analysis of more *S. giganteum* specimens would facilitate the investigation into sexual dimorphism as well as centre of mass, the latter of which would be key in any inquiry into this species' locomotor abilities.

## Supplementary Material

Details of skeletal reconstruction, scaling equations used, and convex hull volumes

## Supplementary Material

Details of fossil specimens

## Data accessibility

Convex hull models are available at Dryad Digital Repository: http://dx.doi.org/10.5061/dryad.83367.
