# Supplementary material for: The extinct, giant giraffid Sivatherium giganteum: skeletal reconstruction and body mass estimation
Source: Biol Lett. 2016 Jan;12(1):20150940. doi: 10.1098/rsbl.2015.0940 (PMC4785933; doi:10.1098/rsbl.2015.0940)
Supplement: Details of skeletal reconstruction, scaling equations used, and convex hull volumes [file rsbl20150940supp1.docx]

Electronic supplementary material

Calculation of femur length

We estimated the unknown *Sivatherium* femur length from the known fossil humerus length, using an ungulate interspecific scaling dataset [1]. The published model uses femur length as the input variable, therefore we have used their raw data to calculate an ordinary least squares linear regression model which uses humerus length as the input variable. Their study investigated the effect of phylogeny on the regression models, and found that phylogeny was not a significant effect in determining scaling patterns.

*Sivatherium* humerus length = 500 mm

**log(femur length) = log(500)*0.9542 + 0.1908**

Estimated femur length = 584 mm

Using *Giraffa* to model the torso of *Sivatherium*

A solution to *Sivatherium’s* missing ribs, non-cervical vertebrae and pelvis is to model them using the entire torso from *Giraffa,* which assumes that thorax dimensions are conserved across Giraffidae; an assumption supported by three observations, detailed below.

1. The proportions of the non-cervical vertebral series are similar in *Giraffa, Okapia* and other non-giraffid ungulates [2].
2. Thoracic circumference scales close to isometry with respect to body mass (exponent of 0.36) in a wide range of bovids*.* This scaling model also accurately predicts the thoracic circumference of *Giraffa*, which tentatively validates its use outside of Bovidae*.*

Circumference and body mass data are taken from [3], which assessed the use of thoracic circumference in predicting body mass across a wide range of bovids. The predictive capacities of the raw and phylogenetically corrected data were similar.

Study [3]’s predictive equation for thoracic circumference and body mass was:

**log(body mass in kg) = 2.711*log(thoracic circumference in cm) – 3.562**

This was based on measurements from a wide range of bovids. If the thoracic circumference (measured at the point of the olecranon) of the giraffe pointcloud (from [4]) is used, it predicts a giraffe body mass of 656 kg. This is very close to (1.028 times) the mass of 638 kg used in study [4].

We used the same dataset to estimate the scaling exponent between thoracic circumference and body mass, using the ‘lmodel2’ package in R. We found that thoracic circumference scales to body mass with an exponent of 0.362 (95% confidence intervals of 0.351 – 0.372), which is close to what is expected with isometry (0.333) but indicates slight positive allometry (Table S1). An isometric relationship would support our method of geometrically scaling up a *Giraffa* torso in order to model the *Sivatherium* torso. In this case, the allometry is so mild, at best, that it leaves us confident that our estimate is reasonable.

| Linear regression method | a | 95% CI of a | b | 95% CI of b | p-value | R^2^ |
| --- | --- | --- | --- | --- | --- | --- |
| OLS | 0.362 | 0.351 – 0.372 | 1.326 | 1.308 – 1.344 | <0.001 | 0.980 |
| MA | 0.362 | 0.352 – 0.373 | 1.325 | 1.308 – 1.342 |  |  |
| SMA | 0.365 | 0.355 – 0.377 | 1.320 | 1.303 – 1.337 |  |  |

**Table S1**, showing linear regression of the log-transformed bovid thoracic circumference (y) and body mass (x), in the form y = ax + b. OLS = ordinary least squares, MA = major axis, SMA = standard major axis. All three methods yield similar scaling exponents.

1. We have compared the thoracic proportions of the same giraffe point cloud, with a 3D model of a mounted adult okapi skeleton (Z2788, Grant Museum of Zoology, University College London). When standardised by length, the corresponding thoracic widths closely agree, indicating that okapi and giraffes have a similarly narrow-profiled thorax.

Ratio of thoracic vertebral series length to thoracic width at the mid-point of rib 7:

Okapi – 1:0.54

Giraffe – 1: 0.51

A notable difference between giraffes and okapi, however, is in their pelvic morphology. Giraffes possess a laterally expanded iliac crest [5] compared to other ruminant artiodactyls, whereas in okapi the iliac crest is more modest in its proportions.

Using *Giraffa* to model the distal phalanges of *Sivatherium*

The *Giraffa* distal phalanx is from the hindlimb of an adult giraffe, with mass of 880kg. This is an uncatalogued specimen kept at the Royal Veterinary College, in JRH’s research collection.

S*ivatherium* minimum convex hull volumes

| Model | Total hull volume (m^3^) |
| --- | --- |
| Initial | 1.022 |
| ‘Minimum’ sensitivity analysis | 0.841 |
| ‘Maximum’ sensitivity analysis | 1.279 |

**Table S2**. Total convex hull volume of the initial model, and the two subsequent sensitivity analysis models. Volumes have been calculated using the ‘Compute geometric measures’ function in Meshlab.

*Sivatherium* mass estimate using convex hull volume

Initial model volume = 1.022 m^3^

Using OLS linear model for log transformed mammal data [6]:

**log(body mass [kg]) = log(volume [m^3^])*0.92 + 3.09**

Using assumed tissue density [4]:

**body mass (kg) = 1.206 * volume m^3^ * 893.36 kg/m^3^**

*Sivatherium* mass estimate using humeral circumference

Minimum circumference = 302 mm

Using circumference vs body mass in mammals >100kg [1]:

**log(body mass [g]) = log(humeral circumference [mm])*2.6488 – 0.0887**

*Sivatherium* mass estimate using thoracic circumference

Thoracic circumference = 338 cm

Using thoracic circumference vs body mass in mammals [3]:

**log(body mass [kg] = log(thoracic circumference [cm])*2.711 – 3.562**

[1] Campione, N.E. & Evans, D.C. 2012 A universal scaling relationship between body mass and proximal limb bone dimensions in quadrupedal terrestrial tetrapods. *BMC Biol* **10**, 60. (doi:10.1186/1741-7007-10-60).

[2] Badlangana, N. 2009 The giraffe cervical vertebral column: a heuristic example in understanding evolutionary processes? *Zoological Journal of the Linnean Society* **155**, 736-757.

[3] De Esteban-Trivigno, S. & Köhler, M. 2011 New equations for body mass estimation in bovids: Testing some procedures when constructing regression functions. *Mammalian Biology - Zeitschrift für Säugetierkunde* **76**, 755-761. (doi:10.1016/j.mambio.2011.07.004).

[4] Sellers, W., Hepworth-Bell, J., Falkingham, P., Bates, K., Brassey, C., Egerton, V. & Manning, P. 2012 Minimum convex hull mass estimations of complete mounted skeletons. *Biology Letters*, rsbl20120263.

[5] Janis, C.M., Theodor, J.M. & Boisvert, B. 2002 Locomotor evolution in camels revisited: a quantitative analysis of pedal anatomy and the acquisition of the pacing gait. *Journal of vertebrate paleontology* **22**, 110-121.

[6] Brassey, C.A. & Sellers, W.I. 2014 Scaling of Convex Hull Volume to Body Mass in Modern Primates, Non-Primate Mammals and Birds. *PloS one* **9**, e91691. (doi:10.1371/journal.pone.0091691).
